# Evidence for a Role of the Host-Specific Flea (*Paraceras melis*) in the Transmission of *Trypanosoma (Megatrypanum) pestanai* to the European Badger

**DOI:** 10.1371/journal.pone.0016977

**Published:** 2011-02-14

**Authors:** Regina Lizundia, Chris Newman, Christina D. Buesching, Daniel Ngugi, Damer Blake, Yung Wa Sin, David W. Macdonald, Alan Wilson, Declan McKeever

**Affiliations:** 1 Royal Veterinary College, University of London, Hatfield, United Kingdom; 2 Wildlife Conservation Research Unit, Department of Zoology, University of Oxford, The Recanati-Kaplan Centre, Abingdon, United Kingdom; University of California Merced, United States of America

## Abstract

We investigated the epidemiology of *Trypanosoma pestanai* infection in European badgers (*Meles meles*) from Wytham Woods (Oxfordshire, UK) to determine prevalence rates and to identify the arthropod vector responsible for transmission. A total of 245 badger blood samples was collected during September and November 2009 and examined by PCR using primers derived from the 18S rRNA of *T. pestanai*. The parasite was detected in blood from 31% of individuals tested. *T. pestanai* was isolated from primary cultures of Wytham badger peripheral blood mononuclear cells and propagated continually *in vitro*. This population was compared with cultures of two geographically distinct isolates of the parasite by amplified fragment length polymorphism (AFLP) and PCR analysis of 18S rDNA and ITS1 sequences. High levels of genotypic polymorphism were observed between the isolates. PCR analysis of badger fleas (*Paraceras melis*) collected from infected individuals at Wytham indicated the presence of *T. pestanai* and this was confirmed by examination of dissected specimens. Wet smears and Giemsa-stained preparations from dissected fleas revealed large numbers of trypanosome-like forms in the hindgut, some of which were undergoing binary fission. We conclude that *P. melis* is the primary vector of *T. pestanai* in European badgers.

## Introduction

A variety of trypanosome species are found in domesticated and free-living British mammalian fauna (Table 1). As stercorarian trypanosomes, all of these parasites undergo development and differentiation within the gut of the arthropod vector and are transmitted to their mammalian hosts contaminatively, through ingestion of the vector or its faeces. Trypanosomes of British cattle (*T. theileri*) and sheep (*T. melophagium*) are classified in the subgenus *Megatrypanum* and are transmitted respectively by tabanid flies [1] and the sheep ked [2]. In contrast, the majority of trypanosomes found in British wild mammals are grouped within the subgenus *Herpetosoma*, of which the rat parasite *T. lewisi* is the type species, and are transmitted by fleas [3]. An exception to this is *T. pestanai*, a parasite of European badgers (*Meles melis*), which is classified in the sub-genus *Megatrypanum*. The arthropod vector of *T. pestanai* is currently unknown. The convention for classification of the Stercoraria has been questioned because it is based largely on morphological parameters and host species [4]. Indeed, a number of analyses at the molecular level have indicated that both the *Herpetosoma* and *Megatrypanum* are polyphyletic [5], [6]. A recent study of the evolutionary relationships of *T. rangeli*, a parasite generally accepted as belonging to the subgenus *Herpetosoma*, concluded that the use of these classifications should be discontinued [5]. Since its first description in Portugal in 1905 [7], *T. pestanai* has been reported in badgers from France [8], England [9] and Ireland [10]. The prevalence of the parasite in a badger population resident in Wytham Woods, Oxfordshire, has been investigated previously through microscopic analysis of blood smears [11] where seasonal and age-related differences were observed. However, interpretation of these observations has been confounded by the lack of information on the transmission vector. A number of blood-feeding ectoparasites are found on badgers, including the flea *Paraceras melis* and tick species such as *Ixodes hexagonus*, *I. ricinus* [12] and *I. canisuga*, although ticks are rarely present on animals trapped at Wytham (C. Newman, unpublished observations). In contrast, *P. melis* is highly prevalent among Wytham badgers - and badgers generally [13] - with some animals experiencing substantial infestations [14]. Given the prominent role of flea species in transmission of *Herpetosoma* trypanosomes of other British wild fauna, these observations present *P. melis* as a compelling candidate vector for *T. pestanai*. We have therefore examined the role of *P. melis* in transmission of *T. pestanai* between badgers, using a PCR-based parasite detection system in association with morphological analysis of fleas collected from PCR^+ve^ badgers. We investigated whether the flea supports development of the insect stages of the parasite which would indicate that it represents the principal transmission vector. The use of PCR techniques also allowed us to extend our previous observations of *T. pestanai* prevalence in Wytham badgers, by achieving higher levels of sensitivity. In addition, we also investigated whether genetic diversity exists between geographically distinct isolates of *T. pestanai*.

**Table 1:** 

Mammalian host	Trypanosome	Sub-genus	Vector	Reference
Wood mouse *(Apodemus sylvaticus)*	*T. grosi*	*Herpetosoma*	Flea	[34]
Bank vole *(Clethrionomys glareolus)*	*T. evotomys*	*Herpetosoma*	Flea	[34], [35]
Rat (*Rattus* sp)	*T. lewisi*	*Herpetosoma*	Flea	[18], [36]
Field vole *(Microtus agrestis)*	*T. microti*	*Herpetosoma*	Flea	[37]
Rabbit (*Oryctolagus cuniculus*)	*T. nabiasi*	*Herpetosoma*	Flea	[38]
European Badger *(Meles meles)*	*T. pestanai*	*Megatrypanum*	Unknown	[7]
Cow *(Bos Taurus)*	*T. theileri*	*Megatrypanum*	Tabanid flies	[1]
Sheep *(Ovis aries)*	*T. melophagium*	*Megatrypanum*	Sheep ked	[2]
Mole (*Talpa europaea*)	*T. talpae*	*Megatrypanum*	Mite	[39]

## Results

### Prevalence of *Trypanosoma pestanai* and dynamics of infection and transmission

In total, 245 blood samples were collected from 207 badgers during trapping sessions in September and November 2009. DNA extracted from each blood sample was analysed by PCR using primers (TPEF1, TPEB1) derived from the 18S rRNA of *T. pestanai*. A product of the expected size (∼513 bp) was amplified in the infected samples. Of the 245 blood samples screened, 78 samples tested positive (31% prevalence) for *T. pestanai*. Of 207 individual badgers analysed, 170 were trapped only once, 36 were trapped twice and 1 was trapped 3 times. The PCR prevalence of *T. pestanai* infection in individual badgers (no repeats) from the first trapping was 29.3%. To study the dynamics of infection and transmission of *T. pestanai*, several parameters were evaluated: trapping session, sex, age, body condition, location (sett) and number of fleas. No significant differences were observed between prevalence rates for the September and November trapping sessions (36% and 28% prevalence respectively, *p* = 0.289, Pearson Chi-Square test). However, the prevalence of *T. pestanai* was significantly higher in males (42%) than in females (27%) (*p* = 0.025, Chi-Square test). Cubs (less than one year old) had significantly higher rates of infection (40% prevalence) than both young adults (1–5 years old, 35% prevalence) and mature adults (more than 5 years old, 16% prevalence) (*p* = 0.04, linear by linear association test). After adjusting for age, evidence for an association between sex and the presence of *T. pestanai* in blood was apparent in a multivariable logistic regression analysis (*p* = 0.033). Males were twice as likely to be infected as females (OR = 1.90; 95% CI: 1.05–3.43). After adjusting for sex in a multivariable logistic regression test, there was evidence for a difference in infection rate between cubs and adults (*p* = 0.04), with adults being more resistant to infection (OR: 0.29; 95% CI: 0.093–0.916). No evidence of association between body condition and prevalence of *T. pestanai* in blood was observed (*p* = 0.563, Linear by linear association test). Similarly, no evidence was found for an association between flea burden and presence of *T. pestanai* in blood (*p* = 0.122, Linear by linear association test). To examine the dynamics of *T. pestanai* infection over time, blood samples from 36 badgers that were caught in both trapping sessions were examined by PCR. Of these, 18 (48%) were negative on both occasions, and 9 (24%) showed persistent infection (or concurrent recrudescence of infection) across trappings. Four badgers observed to be infected in September tested negative in November (10%), while 5 animals that were negative in September had become infected by November (13%). These data are consistent with a cyclical pattern of *T. pestanai* prevalence.

### Isolation of *T. pestanai* and morphological characteristics of axenic cultures

**Figure 1:**
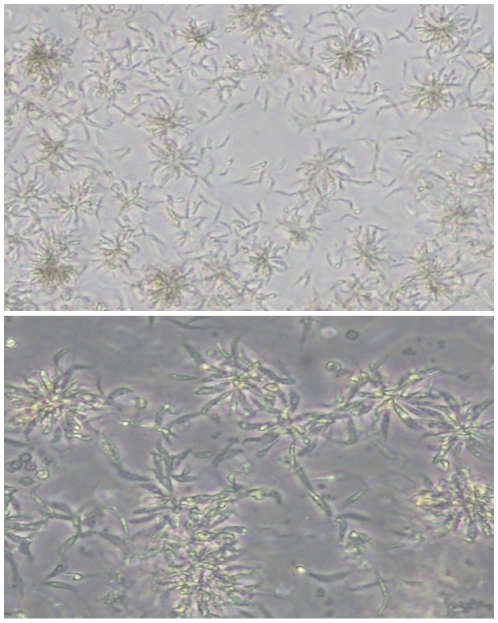
*T. pestanai* (Oxford isolate) in axenic culture. Formation of rosettes as a result of incomplete separation of daughter cells observed by inverted phase contrast microscopy at different magnifications (upper panel, ×20; lower panel, ×40).

**Figure 2:**
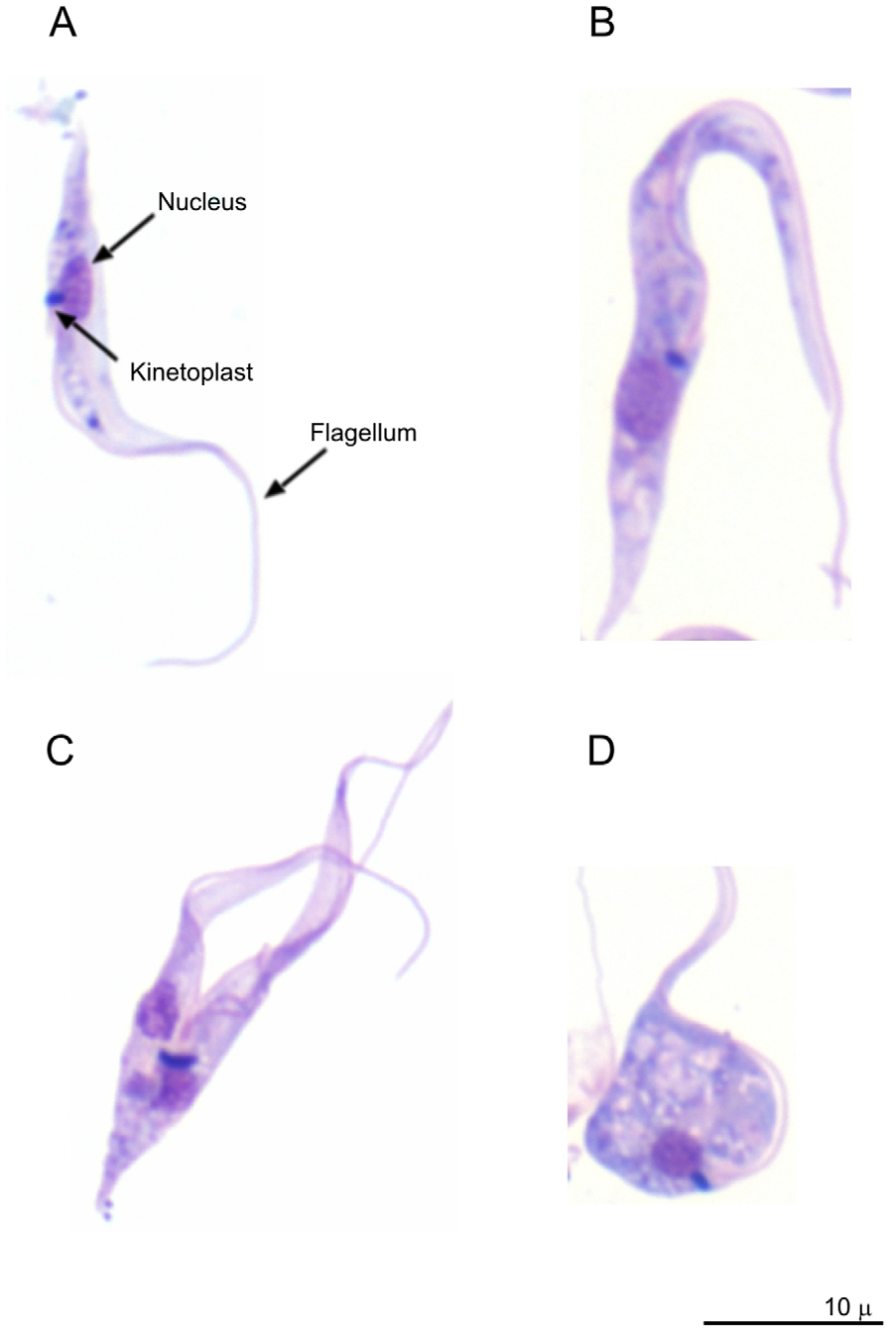
Giemsa-stained smears showing different *T. pestanai* forms in axenic culture. (**A**) Epimastigote-like long slender form. (**B**) Epimastigote-like swollen form. (**C**) Dividing epimastigote. (**D**) Large pear-shaped form (“degenerative” form).

Live motile *T. pestanai* parasites were invariably observed in cultures of peripheral blood mononuclear cells established from PCR^+ve^ blood samples. Moreover, these parasites continued to multiply under the culture conditions used, often giving rise to rosette-like aggregates (Figure 1). Giemsa-stained smears (Figure 2) illustrate characteristic trypanosome features (e.g. kinetoplast and flagellum) observed in cultured *T. pestanai* parasites. A variety of parasite morphologies were observed, including slender (Fig. 2A), broad and intermediate forms (Fig. 2B), and parasites undergoing division/binary fission (Fig. 2C) and degeneration as manifested by transformation to a spherical form with granular degeneration of the protoplasm (Fig. 2D). All three *T. pestanai* isolates (East Anglia, Oxford and France) showed similar morphologies in culture.

### Genetic characterisation of three geographically distinct cultured *T. pestanai* isolates

**Figure 3:**
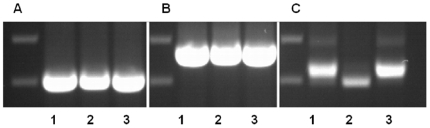
Comparison by PCR of 3 geographically distinct isolates of *T. pestanai*. DNA extracted from different *T. pestanai* isolates in axenic culture (1. East Anglia isolate; 2. Oxford isolate; 3. France isolate) was analysed by PCR using primers derived from the 18S rRNA (A. TPEF1/B1 primers; B. TPEF2/B2 primers) and ITS1 sequences of *T. pestanai*. (C. KIN1/KIN2 primers).

Total DNA extracted from cultures of three geographically distinct *T. pestanai* isolates was analysed by PCR (Fig. 3). PCR analysis using primers derived from the 18S rRNA of *T. pestanai* resulted in amplification of an identical band from all three isolates when using TPEF1/ TPEB1 (Fig. 3A) and TPEF2/TPEB2 (Fig. 3B) primers. However, PCR analysis using primers specific for the ITS1 sequence (KIN1, KIN2) revealed different size bands (Fig. 3C). More detailed genetic characterization using AFLP revealed clear genetic polymorphism between all three isolates (Figure S1). Four selective AFLP primer combinations were used, yielding 56 markers, 41 of which were polymorphic for one or more isolates. The Jaccard index of similarity ranged from 34 to 64%, indicating elevated levels of genetic heterogeneity among the isolates. The highest coefficient of similarity was found between the France and East Anglia isolates (64%), followed by the Oxford and East Anglia isolates (48%) and by the Oxford and France isolates (34%).

### Detection of robust IgG responses of badgers against the parasite

**Figure 4:**
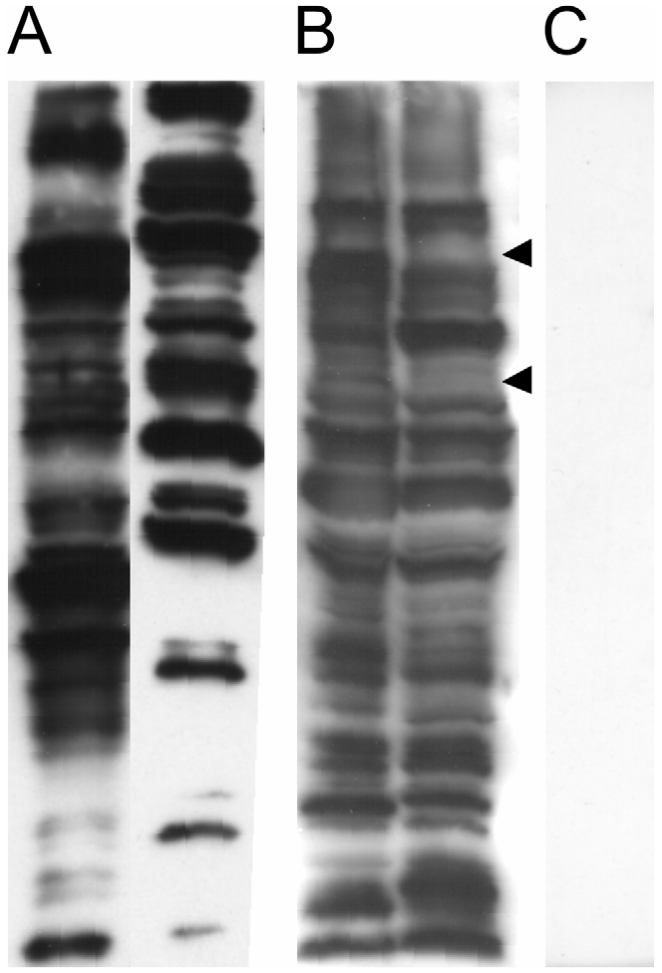
Western blot showing a robust IgG response of badgers against *T. pestanai* lysates. (A, left) Female badger serum, PCR negative in blood. (A, right) male badger serum, PCR positive in blood. (B, left) badger serum response against East Anglia isolate. (B, right) same badger serum response against Oxford isolate. (C) Western blot in the absence of badger serum (HRP-conjugated anti-badger IgG antibody only).

Western blot analysis of sera from infected (PCR^+ve^ in blood) and uninfected (PCR^−ve^ in blood) badgers showed a broadly specific IgG response against *T. pestanai* lysates without exception, with male and female animals showing similar breadth of response (Fig. 4A). Antigenic differences were evident between the Oxford and East Anglia isolates when probed with individual badger sera (Fig. 4B). No seronegative badgers were observed over the trapping period.

### Detection of *T. pestanai* in badger fleas *(P. melis)*

**Figure 5:**
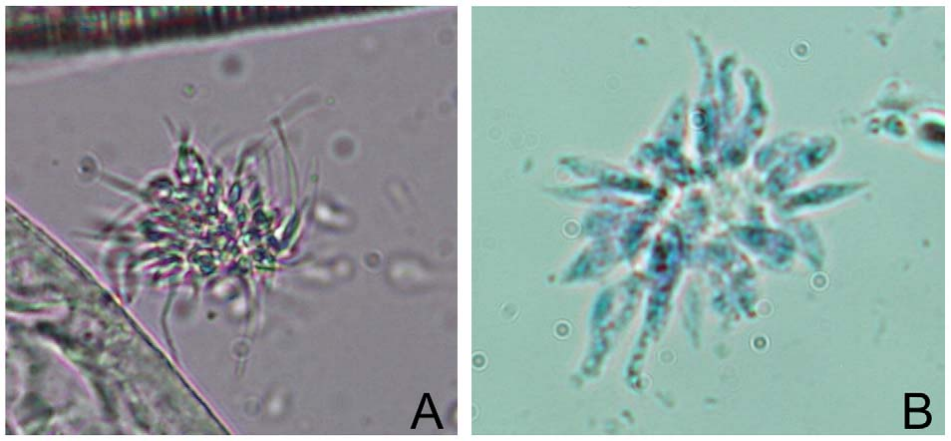
Wet-smear of *T. pestanai* detected in the flea of infected badger. (A) Presence of live motile *T. pestanai* parasites in the hindgut of an infected flea observed by phase contrast microscopy (×40). (B) Detection of rosette in the hindgut of an infected flea observed by phase contrast microscopy (×100).

PCR amplification was carried out on DNA extracted from 26 individual fleas collected from infected badgers (n = 18) and 12 fleas from uninfected individuals (n = 8) using the *T. pestanai*-specific primers TPEF1 and TPEB1. Of those collected from infected badgers, 16 fleas (61%) tested positive for *T. pestanai*, while 2 (16%) of those from uninfected animals yielded a PCR product. The difference in *T. pestanai* PCR prevalence between fleas collected from infected versus uninfected badgers was significant (*p* = 0.015), as revealed by a Fisher's exact test. Similarly, numbers of infected fleas obtained from infected badgers were significantly higher (*p* = 0.026) than those found on uninfected individuals. To confirm that *T. pestanai*-specific PCR products observed in a proportion of fleas reflects development of the parasite within the insect rather than simple contamination of a blood meal, a number of fleas collected from infected badgers were dissected and their gut tissues examined by phase contrast microscopy. In a proportion of these fleas, large numbers of trypanosomes were observed in the hind gut. These were motile and in some cases exhibited the same rosette formation observed in *in vitro* cultures (Fig. 5A and 5B).

## Discussion

Available literature on *T. pestanai* is sparse and, like many of the *Megatrypanum* trypanosomes, the parasite has been classified on the basis of its morphological appearance in blood smears [9]. Criteria applied in this regard include large size, a small kinetoplast located close to the nucleus, and a pointed posterior end. Such parameters are of questionable value where parasitaemias are low and the biology of the parasite is incompletely understood. For example, our measurements of cultured *T. pestanai* indicate an overall length of 25.33–33.33 µm (*n* = 82) whereas Pierce & Neal (1974) recorded values of 25.6–41.4 µm in blood smears. In addition, the extent to which morphological classification reflects true evolutionary relationships is in some doubt. Hence, published information on trypanosome 18S rRNA phylogenies generally places *T. pestanai* apart from *T. theileri*, the type species of subgenus *Megatrypanum* [5], [15], with its closest relative being a species isolated from a wombat. Indeed, *Megatrypanum* trypanosomes fail to form discrete clusters under this type of analysis, distributing instead in apparently unrelated phylogenetic groupings [16]. We now provide compelling evidence that the badger flea *P. melis* is the definitive invertebrate host of *T. pestanai*. This ectoparasite is highly prevalent among badgers from this focal Wytham Woods badger population [11] and its host specificity is consistent with an evolutionary relationship with *T. pestanai*. In particular, badgers exhibit extensive grooming habits to control fleas [17], providing continuous opportunity for ingestion of infected fleas and their faeces. Badgers that tested positive for *T. pestanai* by PCR were consistently found to harbour fleas that also yielded PCR products. This is unlikely to reflect simple carry-over of parasites in the blood meal, given the low parasitaemias observed in badgers [11] and the sensitivity of the PCR system used. Trypanosomes of mammals will be considered under two major groups, Salivarian or Stercorarian, based on whether they undergo anterior station (foregut) development or posterior station (hindgut) development in the insect vector, respectively. Indeed, where trypanosomes were found in dissected fleas from badgers, these were located in the hindgut rather than the foregut and, in many instances, formed rosette-like structures characteristic of replicating *T. pestanai* cultures. These parasites were distinct from crithidial forms and gregarine protists that were also present in a proportion of dissected fleas (data not shown). Precise details of the life cycle of *T. pestanai* in the insect vector remain to be determined. However, by analogy with *T. lewisi*, it is likely that it undergoes a period of cyclic development in the flea to produce metacyclic forms infective for the mammalian host [3] [18]. After ingestion by the flea, trypomastigote forms of *T. lewisi* penetrate the epithelial cells of the stomach of the flea and replicate. Upon rupture of the infected cell, daughter trypomastigotes enter the lumen of the flea stomach and migrate to the hindgut and rectum, where they transform into epimastigotes. These undergo further multiplication, producing large numbers of infective metacyclic forms in the rectum, which are discharged in the faeces. The mammalian host becomes infected contaminatively by ingestion of flea faeces or intact fleas. *Megatrypanum* parasites are transmitted by a diverse range of vectors, including tabanid flies (*T. theileri*), keds ( *T. melophagium*) and mites (*T. talpae*) (see Table 1). Our evidence that *T. pestanai* is transmitted by *P. melis* fleas makes it unique among the *Megatrypanum* trypanosomes and is perhaps indicative of a closer relationship with those parasites currently classified within the subgenus *Herpetosoma*. Although recent phylogenetic analysis based on 18S rRNA and the glyceraldehyde phosphate dehydrogenase (GAPDH) gene [15], [19] are consistent with this possibility, the precise relationships remain unclear. Macdonald *et al.* [11] observed trypanosomes in only 33 (4.6%) of 718 blood smears collected from the Wytham badgers between 1989–1991. Of the 263 badgers examined during that period, only 20 (7.7%) yielded at least one positive test. This was not considered to be an absolute reflection of prevalence, as detection of *T. pestanai* in blood smears is difficult, especially where parasitaemia is low. Indeed, *T. pestanai* was isolated in culture from four blood samples that were negative by blood smear analysis in that study. The PCR-based methodologies used in the present study clearly provide a more reliable and precise diagnostic tool and reveal a considerably higher infection prevalence in the study population. Significantly higher prevalence of *T. pestanai* infection was observed in male badgers than in females. Several host-parasite systems, including those of humans, exhibit sex-related differences [20]. Although either sex can show a higher rate of infection, many studies suggest that females mount a more effective immune response [21], [22], [23]. Testosterone has been observed to enhance susceptibility of rats to *T. cruzi*, as evidenced by higher parasitaemia associated with reduced T cell responsiveness [24]. It is therefore possible that the gender bias observed in *T. pestanai* prevalence among the Wytham badgers relates to a hormonal influence on immunity. However, we observed broad specificity of serum IgG responses in both male and female badgers in immunoblots of *T. pestanai* lysates. Furthermore, studies of the coccidian parasites *Eimeria melis* and *Isospora melis* in the Wytham badger population revealed no evidence that prevalence, and hence immune-susceptibility or risk of exposure to either parasite species varied with gender at any stage of maturity [25], [26]. Given the communal sleeping habits of badgers, and the frequency with which fleas move between hosts [14], we consider it unlikely that the differences in *T. pestanai* prevalence between males and females arises from exposure to the vector. However, Macdonald *et al.* (2008) [27] observed within a high density population of badgers that males move more between groups than do females. Dispersing males tended to move to larger groups and to groups with a preponderance of females. This bias was influenced by season, occurring more in autumn and spring. Our data are derived from material collected from the Wytham badgers during the autumn trapping, when higher rates of movement would be expected among males. It is therefore possible that the male bias in *T. pestanai* infection prevalence relates to badger dispersal and flea exchange. Under these circumstances, males might be expected to pick up fleas from other badger groups, enhancing their potential for infection with *T. pestanai*. We also observed a strong association between *T. pestanai* infection and age, with cubs showing substantially higher prevalence rates than adults. This is likely to arise from exposure to fleas. Cubs spend more time in the sett, where they are exposed to contaminated bedding [12] and as a result of close contact with their mother during suckling and grooming. Our data also indicate that Wytham badgers undergo multiple infections with *T. pestanai* over time and that they can remain infected for prolonged periods. Some individuals remained PCR-positive over two trapping exercises, while others appeared to lose the parasite during the interval between trappings. In addition, the complexity of the patterns observed with badger sera on immunoblots of *T. pestanai* lysate appeared to increase with age (data not shown). Indeed, no seronegative badgers were observed over the trapping. These observations are consistent with a model based on repeated infections that perhaps evolve to a prolonged or intermittent carrier state. Precise resolution of this situation will await further evaluation in the Wytham badgers and identification of appropriate molecular markers. Our preliminary observations with AFLP and the 18S rRNA and ITS1 sequences are consistent with genetic polymorphism between isolates of *T.pestanai*. Indeed, the two UK populations, although moderately disparate in origin (Oxfordshire and East Anglia), are sufficiently diverse to classify as distinct sub-species. Acknowledging that these data are derived from small sample numbers, they do suggest that *T. pestanai* is genotypically diverse. The origins of this diversity are unclear, in the absence of information on sexual recombination in the flea vector, but it may relate to maintenance of the parasite in the face of what appears to be a robust IgG response in infected badgers. Such diversity confounds interpretations of prevalence data based on relatively conserved 18S rRNA sequences. In particular, elucidation of whether persistence of the parasite as revealed by 18S rRNA PCR relates to repeated infection or prolonged carrier status will require a more refined set of genotypic markers.

In conclusion, we have determined the prevalence of *T. pestanai* infection in Wytham Woods badgers using PCR of 18S rRNA sequences. We observe considerably higher prevalence than that previously reported on the basis of blood smear examination. In addition we report higher prevalence in male badgers and in those <1 year old. We provide compelling evidence that *T. pestanai* in this population is transmitted by the badger flea *P. melis* and further show that badgers mount a vigorous antibody response against the parasite. Finally, we reveal that *T. pestanai* parasites are genotypically diverse with substantial variation being evident between isolates derived from relatively adjacent locations. We propose that this diversity is driven in part by the badger immune response.

## Materials and Methods

### 2.1. Ethics Statement

This study was approved by Natural England and carried out under Natural England Licenses, currently 20001537 and Home Office License PPL 30/2318. All trapping and handling procedures were in accord with the UK Animals (Scientific Procedures) Act, 1992, and approved by the institutional ethical review committee.

### 2.2. Study population

All samples were collected between September and November 2009 from a badger population at Wytham Woods; a 424ha mixed semi-natural woodland site, in Oxfordshire, UK (GPS ref: 51:46:26N; 1:19:19W). This population has been studied continuously since the 1970s [28], [29] and currently comprises ca. 220 adults with ca. 50 cubs per year. As part of ongoing long-term monitoring studies, the population is trapped 1–4 times annually using cage-traps baited with peanuts set in the vicinity of each sett. Upon capture, badgers are transported to a central handling facility and sedated by intramuscular injection of 0.2ml ketamine hydrochloride (100 mg ml^−1^) per kg body weight [30]. At first capture (generally as cubs) every individual is marked with a permanent tattoo number in the left inguinal region. For each badger, the location of capture (‘sett’), sex and age is recorded. Morphometric measures recorded include weight (kg), length (tip of snout to base of sacrum - mm) and body-condition, using a subcutaneous fat score originally developed for sheep on a scale of 1 = emaciated to 5 = very good condition. For the present study, the number of fleas was also counted at each capture during a cursory examination over ∼20 sec interval, by parting the fur and examining the badger's back and underside [14]. Blood samples were taken by jugular venipuncture using a potassium-EDTA vacutainer (BD Vacutainer Systems, Plymouth, UK) and stored at 4°C before being processed in the laboratory within 72h of collection. After sampling, badgers were allowed to recover fully before being released at the site of capture later in the same day.

### 2.3. Parasite isolation from blood

Badger peripheral blood mononuclear cells (PBMC) were isolated from uncoagulated blood by Ficoll density gradient separation. Briefly, 3 ml of Ficoll were underlaid with approximately 3 ml of uncoagulated blood. After 30 min centrifugation at 700×g the white blood cell layer was collected carefully and washed twice in Alsever's solution (200×g for 10 min). The PBMC cell pellet was re-suspended in Schneider's medium containing 20% FCS and incubated in 24-well plates at 28°C in a humidified atmosphere of 5% CO_2_ in air.

The *T. pestanai* France-isolate (LEM 110) was kindly provided by Dr. Wendy Gibson (University of Bristol, UK) while *T. pestanai* East Anglia isolate was isolated from a blood sample kindly provided by Dr. Mark A. Chambers (Veterinary Laboratories Agency, Addlestone, Surrey, UK).

### 2.4. DNA extraction

Total DNA was extracted from blood samples (300 µl of whole blood per badger) using the Wizard Genomic DNA Purification kit (Promega) following the manufacturer's recommendations. Total DNA from individual fleas was extracted using a DNeasy blood and tissue kit (Qiagen). Each flea was soaked for 5 min in 200 µl of phosphate buffered saline in a petri-dish and crushed with the plunger of a disposable plastic syringe. After a brief centrifugation (16000×g for 1 min) the supernatant was transferred into a new sterile eppendorf tube containing 20 µl of Proteinase K (Fermentas) and 200 µl of buffer AL (Qiagen). DNA extraction was then carried out in line with the kit instructions.

### 2.5. PCR

Primers designed to target the *T. pestanai* 18S rRNA coding sequence (Accession no: AJ009159; TPE primers) and kinetoplastid ITS1 sequences (KIN) were used for PCR amplification [31].

TPEF1:5′-CCATGCATGCCTCAGAATCACTGC-3

TPEB1: 5′-GGCACTGCCGGCTCTATTTC-3′

TPEF2: 5′-GCAGCGAAAAGAAATAGAGCCGG-3′

TPEB2: 5′-GTTCGTCCTGGTGCGGTCTAA-3′

KIN1: 5′-GCGTTCAAAGATTGGGCAAT-3′

KIN2: 5′-CGCCCGAAAGTTCACC-3′

PCR amplifications were performed in a total volume of 20 µl containing 2 µl of 10× NH_4_ PCR buffer (Bioline), 1 µl of 50 mM MgCl_2_ (Bioline), 0.5 µl of 10 mM dNTP (Bioline), 4 µl of primer mix (Forward and Reverse at 10 pmol each), 0.2 µl (1 unit) of *Taq*DNA polymerase (Bioline), 2 µl of DNA template and 10.3 µl of distilled water. The reaction profile included an initial denaturation step at 94°C for 10 min, followed by 40 cycles of 45 seconds at 94°C, 1 min at 63°C, 1 min at 72°C and a final step of 7 min at 72°C, using a G-STORM thermal cycler. PCR products were electrophoresed for 75 min at 100V in a 1% Tris-acetic acid-EDTA (TAE) agarose gel containing 1× Safeview Nucleic Acid Stain (NBS Biologicals) for visualization. O'GeneRuler 1kb DNA ladder (Fermentas) was used for sizing the DNA fragments in agarose gels.

### 2.6. Parasite protein lysate preparation

*T. pestanai* parasites (5×10^7^) from axenic culture were washed twice in PBS by centrifugation at 700×g for 10 min. The parasite pellet was resuspended in 80 µl of M-PER lysis buffer (Pierce) containing 1× Halt protease/phosphatase inhibitor cocktail (Pierce). The mixture was vortexed and incubated for 20 min at RT. After centrifugation at 16000×g for 15 min, the supernatant was transferred into a new microcentrifuge tube containing 20 µl of 5× Laemmli buffer and boiled at 90°C for 3 min.

### 2.7. Western blotting

15 µl of protein lysate were loaded in wells of a precast 10% polyacrylamide Tris HCl gel (Biorad) and electrophoresed at 30 mA for 1h. Proteins were then transferred to a 0.2-µm nitrocellulose membrane (Amersham Biosciences) at 100V for 60 min. The membrane was incubated in blocking solution (PBS containing 0.05% Tween 20 and 5% milk powder) for 1h at RT followed by overnight incubation with badger serum (1∶100 in blocking solution). Excess antibody was removed by extensive washing in PBS containing 0.05% Tween 20. The membrane was then incubated with HRP-conjugated anti-badger IgG (clone CF2, kindly provided by Dr. Mark Chambers, VLA) diluted 1∶500 in blocking buffer, for 90 min at room temperature. The membranes were washed extensively in PBS 0.05% Tween 20, and bands were visualized using the ECL system (Amersham Biosciences) and a Curix 60 processor (Agfa-Gevaert N.V., Mortsel, Belgium).

### 2.8. Amplified fragment length polymorphism (AFLP)

Approximately 200 ng total *T. pestanai* genomic DNA was digested using the enzymes *Eco* RI and *Mse* I (Fermentas) prior to ligation to adapters as described previously [32], [33]. Primer pairs (MWG Biotech (UK) Ltd.) were based on the adaptor sequences. Pre-amplification was performed using the *Eco* RI associated primer with no selective base and the *Mse* I associated primer with a single selective base (A). Selective amplifications were performed with primers including two selective bases (*Eco* RI primer −AC+*Mse* I primer −AC; *Eco* RI primer −AC+*Mse* I primer −AT; *Eco* RI primer −CG+*Mse* I primer −AC; *Eco* RI primer −CG+*Mse* I primer −AT). The selective *Eco* RI primers were labelled with ^33^PγATP using T4 polynucleotide kinase (Invitrogen). AFLP products were resolved by denaturing polyacrylamide gel electrophoresis (6% acrylamide, UreaGel 6, National Diagnostics) and visualised by autoradiography. Electrophoretic patterns were converted into binary matrices (1 for presence, 0 for absence of a band) and used for calculation of the Jaccard index for each pair-wise comparison [33] (calculated as the number of common bands/the total number of bands ×100, to quantify sampled genetic similarity as a percentage).

### 2.9. Flea dissection and staining

Individual fleas collected from infected and uninfected badgers were dissected under a dissecting-microscope. Each flea was placed on a glass slide in a drop of phosphate buffered saline and transected at the thoraco-abdominal junction with a scalpel blade. The head and thorax were then discarded and the abdomen was immobilised with a fine needle and opened by cutting along the midline of the dorsal tergites with iris scissors. The abdominal contents were then carefully removed with the aid of fine forceps, and the salivary glands, fat body and (in the case of females) uterine tissues were dissected away and discarded. The residual tissues comprising proventriculus, foregut, intestine, malphigian tubules and hindgut were arranged on the slide before covering the preparation with a coverslip. Slides were examined at 100× magnification under phase contrast to identify trypanosome forms and images were obtained using an Olympus CX41 microscope mounted camera (Olympus DP20).

### 2.9. Statistical analysis

Pearson Chi-square test and Fisher's exact test were used to assess associations between categorical variables and prevalence of infection in blood. Linear by linear association Chi-square test was used to assess association between ordinal variables and prevalence of infection in blood. Logistic regression models were employed to estimate the effects of sex, age groups, body condition scores, number of fleas, trapping session and risk of blood infection. Odds ratio (OR) and 95% confidence interval (CI) are presented. All analyses were carried out using Stata 9 software package (StataCorp, Texas, USA).

## Supporting Information

Figure S1 **AFLP fingerprints generated from DNA samples of 3 geographically distinct isolates of** ***T. pestanai*** (1: France isolate; 2: Oxford isolate; 3: East Anglia isolate) with 4 different primer combinations. The selective *Eco*RI (E) primers and *Mse*I (M) primers included two added bases (either −AC, −AT or −CG).
(TIF)
