# Supplementary figures and images for: Evidence for a Role of the Host-Specific Flea (Paraceras melis) in the Transmission of Trypanosoma (Megatrypanum) pestanai to the European Badger
Source: PLoS One. 2011 Feb 14;6(2):e16977. doi: 10.1371/journal.pone.0016977 (PMC3038870; doi:10.1371/journal.pone.0016977)

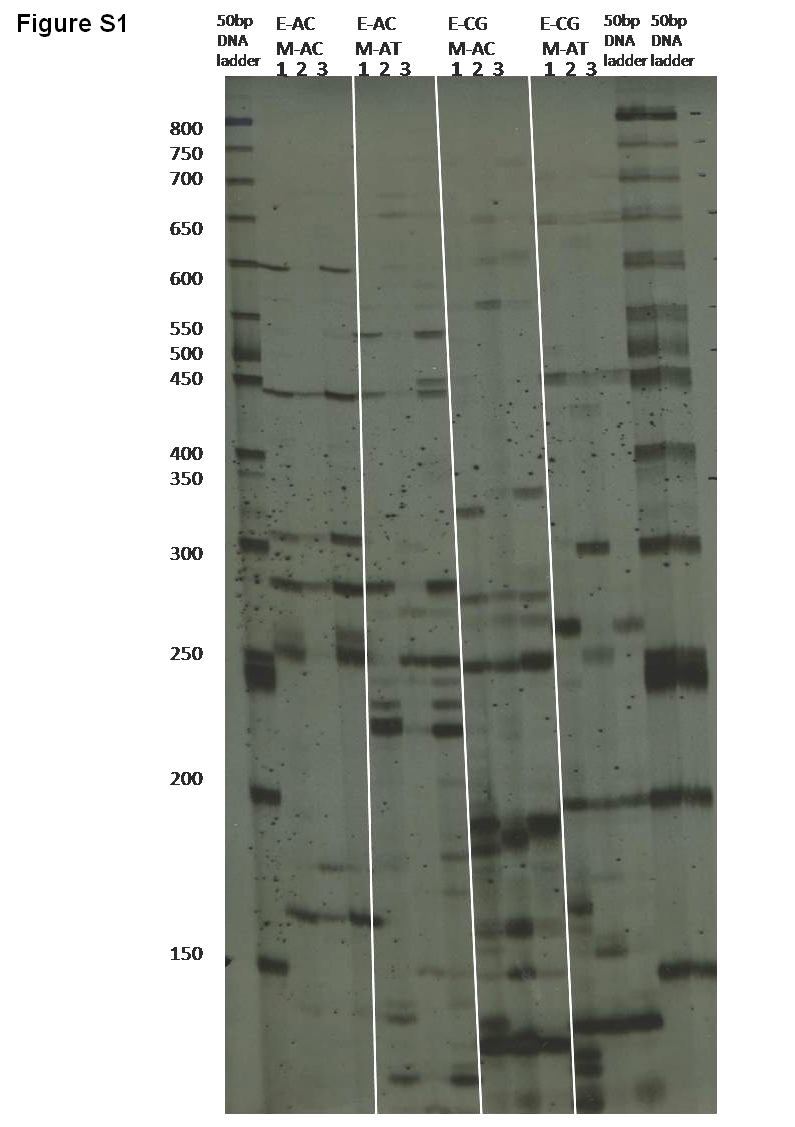

Supplement: Figure S1 — AFLP fingerprints generated from DNA samples of 3 geographically distinct isolates of T. pestanai (1: France isolate; 2: Oxford isolate; 3: East Anglia isolate) with 4 different primer combinations. The selective EcoRI (E) primers and MseI (M) primers included two added bases (either −AC, −AT or −CG). (TIF) [file pone.0016977.s001.tif]
